# Courtwatching: Visibility, publicness, witnessing, and embodiment in legal activism

**DOI:** 10.1111/area.12690

**Published:** 2021-01-28

**Authors:** Nick Gill, Jo Hynes

**Affiliations:** ^1^ Department of Geography University of Exeter Exeter UK

**Keywords:** courts, justice, law, legal geography, trials, tribunals

## Abstract

Courtwatching involves grassroots efforts to observe the day‐to‐day work of decision making in justice systems, usually undertaken by activists as a way to scrutinise and challenge the power of legal professionals such as judges. This paper argues for closer attention to courtwatching in legal geographical research. Numerous courtwatching programmes exist around the world, and the first part of the paper surveys some of these, giving a sense of their diversity, the challenges they can face, and the influence that they have. The second part of the paper uses courtwatching to explore questions of visibility, publicness, witnessing, and embodiment in legal research into courts, trials, and hearings. It argues that courtwatching highlights the complexity of legal publicness, problematising the binary notion of “closed” or “open” hearings, and also raises important questions about the ethical differences between watching and witnessing. Finally, in the context of proliferating ways in which courts are becoming public via digital means of watching, such as TV and podcasts, the paper asks what difference it makes to actually be there, in the flesh, to watch legal processes.

## Abstract 2

The paper sets out what courtwatching is and gives some examples from various countries. It argues that a closer engagement with courtwatching in legal geography will yield insights into the issues of visibility, publicness, witnessing, and embodiment that surround court observations.

## 1 INTRODUCTION

A still dominant body of doctrinal law implicitly assumes that the law is placeless – objective and administered from nowhere by a neutral actor unaffected by their own place in the world (Bartel et al., 2013). Countering this view is central to critical legal studies, including work on the law inspired by legal geography, feminism, post‐colonialism, and queer‐theory (Davies, 2017). This work recognises that the place, race, gender, and class position of law makers and judges matters to how law is experienced, as does the position of legal subjects. In short, law cannot be easily separated from the material conditions of its enactment, and courts are a primary site at which this occurs. Consequently, although legal geographers have tended not to spend as much time in courts as socio‐legal scholars and legal anthropologists, Jeffrey (2019a) has recently called for a more serious engagement with courts and tribunals in legal geography.

Our interest here is courtwatching, meaning grassroots efforts to observe the daily machinery of justice systems and challenge entrenched legal practices or assumptions about them. These objectives are pursued both through courtwatchers’ presence in courts and through their reporting on what they have seen. We distinguish courtwatching from more formal systems of monitoring administered by governments, NGOs, or international organisations.^1^ Rather, courtwatching programmes are generally driven by activists or activist‐researchers with a particular concern about the operation of certain legal systems or decision‐making processes and tend to be coordinated outside large, established organisations and channels of power.^2^

In this paper we argue that attending to courtwatching can contribute to legal geography in at least three ways: by opening up or providing fresh impetus to important questions about the publicness of court space and legal processes; by raising or reigniting questions about the ethics and power of witnessing legal practice; and by pointing towards the contested role of embodiment in legal processes. Legal ethnographers have wrestled with a variety of these issues in their work (e.g., Dahlberg, 2009; Roach Anleu et al., 2016) and we see numerous crossovers between legal ethnography and courtwatching. The focus on courtwatching as an activist practice, however, connects the paper to struggles over justice systems outside the academy, and highlights how urgent and practically relevant scholarly reflections on these issues are.

We begin by setting out some examples of courtwatching in various countries and reflecting on the variety of aims and methods employed by courtwatching programmes. We then reflect on courtwatching through the lenses of visibility/publicness, witnessing, and embodiment before drawing out some conclusions.

## 2 LOCATION AND DIVERSITY OF COURTWATCHING PROGRAMMES

In 1924, Lord Chief Justice Hewart remarked that “justice should not only be done, but should manifestly and undoubtedly be seen to be done.”^3^ This concern with the visibility and transparency of legal processes has long underpinned fundamental values of justice systems, including natural justice and judicial independence. Courtwatching programmes, while varied, continue in this tradition by highlighting the importance of visible legal processes.

Courtwatching has been taking place in the USA for at least the past five decades. Winsor and Dunn (1973) discuss a courtwatching project in Massachusetts that started in 1971 that, they suggest, was part of a wider popular movement at the time aiming “to aerate and demythologize our institutions” (1973, p. 111). They suggest that many of the courtwatching projects at the time were mainly “atmospheric,” predicated on the hope that middle‐class courtwatchers would “tone down the verbal brutality with which defendants were treated” (1973, p. 111). This required a certain visibility of the watchers: one group of female courtwatchers in Louisiana wore white gloves as their trademark, for instance. Some of the largest and most established courtwatching programmes still take place in the USA, often having a connection to a university to help with funding, volunteer recruitment, and data analysis.

Although sometimes seen primarily as a method to aid the “legal‐socialization” of both law and non‐law students (Lindahl, 2007, p. 145), American courtwatching programmes also frequently focus on holding specific elected individuals, such as prosecutors, to account for their election promises in the context of high rates of incarceration of black and Hispanic people (Gilmore, 1999; Loyd et al., 2012; Pfaff, 2017). Observation in the criminal courts is therefore particularly common.^4^

Outside of the USA, a courtwatching project in Bulgaria was established in 2006, with the aim of improving the implementation of new anti‐domestic violence laws. Project members argue that their presence at court hearings “reduces the incidence of bias and discrimination” and increases the likelihood of an immediate protection order being granted (European Institute for Gender Equality, 2015). Their work illustrates the potential of courtwatching to improve public awareness about domestic violence and to support women either experiencing or at risk of abuse.

In the UK, the Bail Observation Project undertakes observations of immigration bail cases as a way to hold immigration judges to account.^5^ In England, they gather data to monitor both the adherence of immigration judges to guidelines^6^ and the rolling out of procedural reforms^7^, especially video link technology, and use this data to lobby for change (Rowe et al., 2019). In Scotland, a collaboration between the University of Glasgow's School of Law and the student‐run Law in Action Group resulted in a comprehensive guide for cautioners^8^ to help them prepare for a bail hearing, which was informed by the students' courtwatching observations (Ferguson & Paul, 2017).

In France, a group of courtwatchers aim to provide “civilian oversight of counter‐terrorism legislations and practice” (Sulzer, 2019). They seek to help inform the practice of human rights actors and legal practitioners. In particular, they see court observation as a way to improve legal transparency in light of a lack of publicly available transcripts or final decisions of counter‐terrorism hearings.

Since their foundation in 2010, Court Watch Poland supports court reform in the Polish justice system by running a programme of “citizen court monitoring” (Pilitowski & Burdziej, 2013, p. 8). This initiative aims to “reverse the increasing alienation of the judiciary from the society” (2013, p. 7) and has a dual mission of court reform and improving civic engagement. Although a common criticism levelled at layperson‐run courtwatching programmes is that people without legal training will not sufficiently understand the proceedings, they argue that lay observers are “more likely to see the problematic nature of some well‐established practices” (2013, p. 11).

This selection of courtwatching programmes demonstrates the range of motivations courtwatching programmes have while maintaining their commitment to using lay in‐person observations to open up court proceedings to a wider audience. However, this selection is not exhaustive. Our discussion is selective as it is based on English‐language searches and available English‐language sources: grassroots courtwatching programmes undoubtedly occur in other court systems worldwide. Moreover, we focus on programmes that pursue progressive ends, yet not all courtwatching programmes will have such goals. There is evidence of increasing interest in the courts among far‐right groups in the UK, for example (BBC News, 2019).

Our limited survey of courtwatching practices nevertheless indicates the diversity of objectives that courtwatchers can pursue. These varying and sometimes productively overlapping objectives are reflected in the courtwatching models proposed by the Community Justice Exchange (2019): exploratory research, civic engagement, individual support, accountability campaigning, advocacy campaigning, and system monitoring.

Enquiries into how successful courtwatching programmes are must be sensitive to this diversity, but by way of indication, Ford (2010) writes evocatively about the power of courtwatching to effect change. The courtwatch she reports on involved 200 volunteers, 199 of whom were women, who observed the family courts in Houston, Texas, ultimately driving a “group of powerful and entrenched family court judges off the bench” and resulting in significant reform (2010, p. 1). For over a decade, charges of corruption had been mounting against several judges at Harris County Family Court. Their story offers a model for other “communities struggling against judicial systems originally meant for their protection that have since gone astray” (2010, p. 3).

The value of all the courtwatching practices surveyed lies in their ability to record and make sense of the “dense spaces” of courtrooms (Faria et al., 2020, p. 1). Combining the material and immaterial (Jeffrey, 2019a), presences and absences (Gill et al., 2019), and the everyday and the structural (Faria et al., 2020), we can think about the space of the courtroom as an “affective arrangement” (Bens, 2018). Capturing the full complexity of these spatial dynamics, whether for activist or activist‐scholarship purposes, is facilitated by the co‐presence of watchers with judges and litigants that court observations entail.

Despite this clear value, we would argue that there should be limits on what courtwatchers can see. Some courts should be private, for instance, when witnesses or juries might be affected by publicity or by the presence of antagonistic groups in the public gallery (Jaconelli, 2002). Furthermore, the assurance of anonymity for witnesses may be an essential part of securing their trust and participation, particularly in cases involving traumatic crimes or war crimes only recently committed (see Jeffrey, 2019b). Closed courts or reporting restrictions may, then, take precedence over open justice in facilitating a fair hearing.

Having set out the diversity of courtwatching programmes, we now consider the contact points between human geographical perspectives and courtwatching. We contend that human geography can offer innovative perspectives on courtwatching practice, and also that courtwatching opens up interesting lines of enquiry for geographers.

## 3 VISIBILITY AND PUBLICNESS

The first contact point concerns the publicness and visibility of court proceedings. Among the courtwatching programmes described, a variety of practical challenges of access were encountered: from being flatly denied access, which can occur at the discretion of judges even if formal rules proscribe public access (Pilitowski & Burdziej, 2013; Stevens, 2009), to bans on note taking (Winsor & Dunn, 1973) and lack of access to court lists (European Institute for Gender Equality, 2015). Other problems included being required or expected by court officials to adhere to principles of “objectivity,” “non‐intervention,” “cooperation,” and “confidentiality,” which courtwatchers can experience as stifling (Richmond, 2012). Courts themselves are also sometimes hard to physically access, being located in remote places, including on islands and within detention facilities.

These challenges highlight the complexity of “publicness” with respect to legal hearings. The outcomes of cases, for example, are sometimes made publicly available online, on a physical noticeboard in a specific court,^9^ or only for a fee via an application to the court in question.^10^ Court security functions to regulate the admittance of observers and their artefacts to the courts according to various, sometimes gendered, norms such as the inadmissibility of mirrors, laptops and tablets, maps and make‐up. Observers’ practical abilities to observe and understand hearings also depend on the speed of the hearing and speech in the court (Hambly & Gill, 2020), the degree of legal jargon used, and the discretionary potential of a range of figures involved in hearings (the judge, solicitors, ushers, and security personnel) to explain what is happening. Even the hardness of the chairs in the public seating areas of courts can act as an effective limit to publicness over the course of a long hearing or series of hearings in the same room.

Geographers have a long‐standing interest in publicness, as well as the constraints placed on public space (e.g., Mitchell, 2003). The practicalities of courtwatching highlight that the notion of a binary between public and closed hearings that is common in public‐facing legal discourse is insufficient to capture the complexities of legal public space in practice. These extend, at least, to issues of access, remoteness, procedure, speech, audibility, comfort, and technology, each of which can be differentially experienced according to differences in class, gender, race, age, and the specifics of the litigant in question. Furthermore, the binary between public and closed hearings espoused by public‐facing legal discourse also insufficiently reflects legal reality and the nuanced internal discussions within law about the openness or otherwise of legal processes. By thinking about public space in multiple and relational terms, geographers have the potential to productively complicate these legal discourses of publicness.

## 4 WITNESSING

Geographers and others have reflected critically on what we might understand by “witnessing.” There has been a reaction, for example, against witnessing that involves only passive watching, particularly in the context of witnessing human rights abuses. Kurasawa (2009) suggests that this passivity or inactivity is linked to various “perils of testimony,” such as silence, indifference, and easily forgetting about what has been witnessed afterwards. Boltanski similarly draws on Arendt's notion of the “politics of pity” to highlight that simple observation of “the *unfortunate* by those who do not share their suffering” (1999, p. 3) constitutes a politics of pity rather than a politics of justice. In her discussion of the ethical problems surrounding recounting testimonies of witnessing state terror, Pratt (2008) highlights the related risks of voyeurism and the “pornography of horror” that occurs when witnesses produce testimony “for the sake of the moral satisfaction of the liberal gaze” (Pratt, 2008, p. 755, quoting Bernstein, 2004, p. 11). Conceptual work on witnessing and testimony has also critiqued the suggestion that written or oral testimony can convey the realities of experiencing state power and state terror (Agamben, 1999), being mindful that body language and comportment can capture elements of this reality that language cannot (Bernald‐Donals & Glesjer, 2001; Dewsbury, 2003; Givoni, 2014).

Faced with these challenges, scholarly literature recommends a range of active approaches to witnessing. Kurasawa (2009) discusses raising one’s voice against atrocities, undertaking practices of empathy and remembrance, and taking measures to ensure future prevention, while Boltanski (1999) emphasises the importance of introducing self‐reflexivity in witnesses’ accounts. A witness cannot be a “simple reporter” (1999, p. 43) describing objectively what they have seen, he argues. Rather their account must include a description of how the witnessing affected the observer. This moves the observer from a position of a simple spectator towards an “*introspector*,” a position from which the witness can incorporate denunciation and anger into their accounts (1999, p. 43). Pratt (2008) discusses the importance of how witnessing is framed, noting the effectiveness of framing witnessing as people‐to‐people solidarity rather than “care” or “duty” (Pratt, 2008). Tait (2011) similiarly draws a distinction between bearing witness and eye witnessing, emphasising that the former entails an active taking up of responsibility in reaction to what has been witnessed.

Various courtwatching programmes are driven, at least in part, by an explicit or implicit desire to witness proceedings (see, for example, Khan, 2014). Despite the potential passivity that the nomenclature of “watchers” imparts, their work often embodies both a sensitivity to the practical and ethical limitations of witnessing, and the utilisation of several of the principles recommended by these scholars to avoid its ethical perils. For example, strong emphasis is often placed on the fact that courtwatching is part of a broader campaign to deconstruct unjust institutions and mechanisms of power. Courtwatching campaigns also routinely have some form of feedback to legal authorities as part of their core objectives, insuring against the “silence” of which Kurasawa (2009) warns. It is this connection between the moment of witnessing and their broader advocacy campaigning that allows courtwatching programmes to adopt more active witnessing styles.

This is not to say that the witnessing involved in courtwatching is uncomplicated. One view of court processes is that they should be impervious to whoever watches them, otherwise they could be influenced by any group organised enough to undertake observations. The imperative of the witnesser to change what they have seen is potentially at odds with the autonomy of courts.

The majority of court‐watching programmes we have reviewed, however, tend to use the information derived from their observations to inform court authorities and suggest improvements to practice. Although this can be a challenging and difficult conversation, it is usually intended as constructive, and court authorities can value the data and consequent suggestions very highly. Court authorities are usually at liberty to take or leave the data and suggestions that courtwatchers collect and make, so there is little threat to judicial independence.

## 5 EMBODIMENT

The embodied nature of court hearings has traditionally generated embodied courtwatching programmes.^11^ Yet, technological innovations such as televised or podcasted court hearings and Twitter feeds from courts can open court processes to public scrutiny in new ways.^12^ Via these technologies, disembodied watching becomes possible, which could facilitate more ambitious, large‐scale courtwatching projects. The move to remote hearings prompted by the COVID‐19 pandemic may open up further such opportunities. However, it is important to question the effect that a separation between the visibility of the court to observers and the embodied presence of watchers might have over legal participation, including observation.

The issue turns on whether being there is indispensable to certain forms of watching. Electronic forms of court publicness such as these usually allow for only a one‐way form of public presence (i.e., observation without the observed being able to identify the watcher). The courtwatching objectives of trying to influence the “atmosphere” in hearings, making judges and other actors aware that they are being observed, or showing solidarity with litigants, are clearly affected by the anonymity and non‐presence of the watcher via these forms of electronically mediated observation. How, for instance, can an invisible observer, possibly watching a recording of a hearing after the event itself, convey solidarity with a litigant who is experiencing a questionable standard of justice or let a judge know that their behaviour in the courtroom has not gone un‐noticed?^13^ Although some commentators, who understand courtwatching to be primarily about following cases through courts and reporting on them, might suggest that physical attendance is not central to courtwatching (Metzmeier, 2008), we argue that being there and being seen is central to at least some of the courtwatching activities outlined above. Witnessing, as Pratt argues, is “fully embodied” (2008, p. 754).

The fact that judicial power and state power are experienced in such an intimate way in courts helps to support the case that an embodied approach is likely to be powerful in pursuing courtwatching objectives. As Jeffrey notes, bodies and law are often “intricately intertwined” (2020, p. 1004). What is more, courts often require the presence of (“the body” of) the litigant under the law of *habeas corpus*:^14^ courts claim, sometimes violently, jurisdiction over the corporeal realm. And yet, often, scant attention is paid to the conditions of “the body” – the health, comfort, ability, alertness, and comprehension of the litigant during hearings (Gill et al., 2019). Feminist geographers have recently drawn attention to the productivity of the lens of the intimate as a way to make visible forms of power and resistance that lie outside traditional dualisms in geography such as global–local and body–world (Little, 2019; Pain, 2015; Pain & Smith, 2008; Pratt & Rosner, 2006). The potential of this lens lies in its ability to make connections between specific instances and sites of power on the one hand, and broader, sometimes global injustices on the other. In a court context this involves both “attend[ing] to the details of the close‐up” (Faria et al., 2020, p. 10) and simultaneously maintaining “a sensitivity to the wider operations of power” (2020, p. 10) at play in the courtroom setting. We would argue that the intimacy of courtwatching in the flesh – hearing the same sounds, experiencing the same atmospheres, feeling the same heat and cold as litigants – can equip courtwatchers with a heightened empathy with experiences of state power. Intimate and embodied approaches to courtwatching help to reclaim and hold accountable the corporeal power that courts claim.

## 6 CONCLUSION

Courtwatching has a varied history, and opens up a number of interesting lines of enquiry for geography. Courtwatching sets the concepts of visibility, publicness, witnessing, and embodiment that ethnographers sometimes discuss within the context of ongoing activist struggles over rights and due processes, bestowing urgency and relevance onto questions about the roles and limitations of these concepts in practice, as well as contributing to scholarly understanding of them. What is more, as justice systems become increasingly privatised and digitised (see Hynes et al., 2020), courtwatching organisations offer a distinctive perspective on debates around open justice, democracy, and state accountability.

By foregrounding how observable court hearings are to the public, courtwatching highlights the complex ways publicness plays out and thus challenges the notion that hearings are either “open” or “closed.” Courtwatching also reiterates important questions about the role of witnessing, its limited ability to fully represent suffering and also the ethical role of the observer. Furthermore, by paying attention to the site of the body, courtwatching can help us to draw attention to the ways in which legal processes are affective, intimate, and embodied. As such, it offers a valuable way into engaging with Jeffrey’s (2019a) call to engage with the material enactment of law in courts and tribunals.

For geographers engaged in courtroom ethnographies, courtwatching productively complicates the positionality of the researcher, and challenges us to consider whether and how our work constitutes “active” witnessing. For some of us, who consider ourselves activists as well as (and through) our scholarship, these concerns are more than simply academic. In this case courtwatching with groups outside of the academy and disseminating insights from our work to courtwatchers may offer opportunities for solidarity building and activist‐scholarship work.

## Data Availability

This paper is not strictly an empirical paper and therefore does not draw on data that is ours to share.

## NOTES

^1^
This is not to dismiss international organisations in the work of “watching” courts, but rather to suggest that formalised monitoring programmes, for example those conducted by the Office of the United Nations’ High Commissioner for Human Rights (2011) to document human rights abuses or improvements, are distinct from the courtwatching methodologies we focus on here.

^2^
As a result of their location outside formal structures of power, courtwatching programmes often face serious challenges of implementation, including shortages of volunteers and resources, difficulties of standardising data collection across multiple individuals in dispersed sites, and difficulties in securing actual or perceived objectivity (McCoy & Jahic, 2006).

^3^
R v Sussex Justices; Ex parte McCarthy [1924] 1 KB 256, 259.

^4^
See, for example, Courtwatch MA (Courtwatch M.A., 2019) and Courtwatch NYC (Schwarzapfel, 2018).

^5^
In the UK, individuals detained under immigration powers may apply for temporary release (“bail”).

^6^
Guidance is produced by the relevant Tribunal for immigration judges. This guidance is neither exhaustive nor binding, but suggests good practice for immigration judges to follow in a bail hearing.

^7^
In particular, a reform programme that has been underway since 2017 to digitise many of the processes in courts and tribunals in England and Wales.

^8^
Also called sureties or financial conditions supporters.

^9^
For example, in France’s asylum court until recently.

^10^
For example, in various German courts dealing with asylum appeals.

^11^
Not all court hearings are now embodied or in‐person processes, with an emerging trend developing towards remote hearings. We do not consider the challenges and opportunities of disembodied watching of disembodied court hearings here, although consider this an interesting future avenue of research.

^12^
Although, see Resnik (2006, 2019) for reservations about online courts and publicness.

^13^
On the other hand, it might be argued that if the judge knows there is a possibility that they are being watched via electronic means, courtwatchers’ presence may be felt more pervasively, along the lines of Foucault’s panopticon.

^14^
Habeas corpus means, literally, “that you have the body.”
